# Prognostic Value of Programmed Cell Death Ligand 1 (PD-L1) Expression in Patients With Papillary Thyroid Carcinoma: A Retrospective Biomarker Study

**DOI:** 10.7759/cureus.91281

**Published:** 2025-08-30

**Authors:** Dimitra Spyroulia, Dimitrios Lefantzis, Spyros Lygeros, Eirini Nikolaou, Stylianos Triantos, Dimitrios Theodosiou, Vassilis Samaras, Pavlos Maragkoudakis

**Affiliations:** 1 Department of Otolaryngology, Korgialenio-Benakio Hellenic Red Cross Hospital, Athens, GRC; 2 Department of Otolaryngology – Head and Neck Surgery, University Hospital of Patras, Patras, GRC; 3 Chemical Engineering, Kallifronas S.A., Athens, GRC; 4 Department of Pathology, Korgialenio-Benakio Hellenic Red Cross Hospital, Athens, GRC; 5 2nd Department of Otolaryngology, Attikon University Hospital, National and Kapodistrian University of Athens, Athens, GRC

**Keywords:** clinicopathological marker, immune checkpoint, immunohistochemistry, papillary thyroid carcinoma, pd-l1, prognostic biomarker, programmed death-ligand 1, thyroid cancer, tumor immune microenvironment

## Abstract

Background: Programmed cell death ligand 1 (PD-L1) expression is a well-established prerequisite for the use of immune checkpoint inhibitors and is increasingly recognized as a prognostic biomarker in various malignancies. However, its prognostic significance in thyroid cancer, particularly papillary thyroid carcinoma (PTC), remains largely unexplored and somewhat controversial, with findings to date being limited and inconsistent. The aim of this study was to evaluate PD-L1 expression in a well-characterized cohort of patients with PTC and assess its association with key clinicopathological features and clinical outcomes.

Materials and methods: This retrospective, single-center cohort study included patients with histologically confirmed PTC who underwent thyroid surgery at the Korgialenio-Benakio Hellenic Red Cross Hospital of Athens in Greece between 2007 and 2018. Formalin-fixed, paraffin-embedded tumor samples were assessed for PD-L1 expression using immunohistochemistry and scored using the combined positive score (CPS), with CPS ≥10 indicating PD-L1 positivity. Clinicopathological data were collected through review of patients’ medical records and postoperative histopathology reports, and associations with PD-L1 status were analyzed. Disease-free survival (DFS) and overall survival (OS) were also evaluated using Kaplan-Meier methods.

Results: Among 81 patients with PTC included in this study (median age: 48 years, 61/81 (83%) patients being female), PD-L1 positivity (CPS ≥10) was observed in 37/81 (45.7%) patients. CPS ≥10 status was significantly associated with larger tumor size, higher pT stage (pT3-pT4, as per the 8th edition of the American Joint Committee on Cancer (AJCC) tumor-node-metastasis (TNM) classification), extrathyroidal extension, lymph node metastasis, and the presence of aggressive histologic variants. Coexisting Hashimoto’s thyroiditis and higher rates of radioactive iodine therapy were also more common in the PD-L1-positive group. At a median follow-up of nine years, the overall recurrence rate was 8.6% (7/81 patients), with significantly higher recurrence and shorter DFS among patients with CPS ≥10. OS remained high across both groups.

Conclusions: PD-L1 expression was present in nearly half of this real-world Greek PTC cohort and was associated with multiple adverse clinicopathological features and an increased risk of recurrence. These findings support the potential utility of PD-L1 as a prognostic biomarker in PTC and may inform postoperative risk stratification and follow-up strategies, although further prospective studies in larger, multicenter cohorts are warranted to corroborate these results.

## Introduction

Thyroid cancer (TC) is the most common malignancy of the endocrine system. It ranks as the seventh most commonly diagnosed cancer worldwide, with over 821,000 new cases annually (4.1% of all cancer cases) [1]. It disproportionately affects women with a female-to-male ratio of approximately 3:1 and is often diagnosed at a younger age compared to any other adult cancer [1,2]. Over the past decades, the global incidence of TC has been dramatically increasing, particularly in high- and middle-income countries, although the magnitude of this rise varies across and within countries [3,4]. Conversely, mortality rates have remained stable or even declined, now converging toward similarly low levels across most countries [5].

This surge in TC incidence has been largely driven by papillary thyroid carcinoma (PTC), which represents the predominant histological type, accounting for nearly 90% of all TC cases [3,6]. Although PTC typically follows an indolent course and features an excellent prognosis, reflected in a five-year survival rate of up to 99.7% when detected early and treated with standard surgery, radioactive iodine (RAI), and/or thyroid hormone replacement therapy, a subset of patients still develops local recurrence or distant metastasis, resulting in an unfavorable prognosis and even death [7,8]. Despite the utilization of established clinicopathologic factors to refine recurrence risk stratification, such as lymph node involvement, multifocality, histological variant, extrathyroidal extension, and vascular invasion, outcomes can still vary considerably among patients within the same risk group. This heterogeneity complicates clinical management and underscores the need for improved prognostic tools, including emerging molecular biomarkers that reflect tumor aggressiveness, to more accurately stratify risk and guide management in TC [9].

Interestingly, a growing body of evidence highlights the tumor immune microenvironment as a key driver of tumor progression. In fact, tumors can shape their immune microenvironment to create an immunosuppressive milieu that allows them to evade immune surveillance, primarily by exploiting immune checkpoint pathways such as the programmed cell death 1 (PD-1)/programmed cell death ligand 1 (PD-L1) axis [10]. PD-1 is a transmembrane receptor expressed on activated T cells, while its ligand, PD-L1, is expressed on tumor cells and various immune cells. The binding of PD-L1 to PD-1 inhibits T cell proliferation and cytokine production, leading to T cell exhaustion and tumor immune evasion, now recognized as one of the hallmarks of cancer and the biological foundation of contemporary immunotherapy [9-11]. This understanding has led to the development and approval of monoclonal antibodies targeting the PD-1/PD-L1 pathway, which have transformed the therapeutic landscape of several difficult-to-treat malignancies, including melanoma, non-small cell lung cancer, renal cell carcinoma, and bladder and gastric cancers, where PD-L1 often serves as a predictive and prognostic biomarker [9].

In TC, PD-L1 has also been evolving as a potential prognostic biomarker [11]. Several recent studies have investigated PD-L1 overexpression across histologic subtypes of TC, particularly in PTC, with many reporting associations with adverse clinicopathological features such as larger tumor size, lymph node metastasis, multifocality, extrathyroidal extension, thyroiditis, and the presence of the BRAFV600E mutation. However, some studies have not confirmed such associations [7,9]. Meta-analyses further support a significant association between PD-L1 overexpression and poor survival outcomes, as well as a higher risk of recurrence, suggesting its potential role in risk stratification and guiding immunotherapy [12-14]. Nonetheless, the current evidence remains inconclusive, and a consensus has yet to be reached, largely due to marked variability in PD-L1 expression levels reported across studies. This variability is primarily driven by differences in the populations studied and methodological inconsistencies in immunohistochemical (IHC) protocols, including the use of different PD-L1 antibody clones, tissue preparation methods, scoring systems, and cut-offs to determine positivity [7,9]. As a result, the prognostic relevance and clinical utility of PD-L1 in PTC remain uncertain and warrant further investigation.

Therefore, the aim of our study was to evaluate PD-L1 expression in a Greek cohort of patients with PTC and assess its association with key clinicopathological characteristics. By further investigating this relationship, we sought to explore the potential of PD-L1 as a prognostic biomarker in PTC.

## Materials and methods

Study design and patient population

This was a retrospective, single-center cohort study of adult patients (aged ≥18 years) with a histopathologically confirmed diagnosis of PTC following thyroid surgery at the Korgialenio-Benakio Hellenic Red Cross Hospital of Athens in Greece between 2007 and 2018, ensuring a minimum of five years of follow-up at the time of analysis. Eligible patients had a primary tumor size ≥0.5 cm and adequate clinical and histopathological data, defined as complete documentation of the key variables pertinent to the study objectives, as described below. No exclusion criteria were applied, and patients were not excluded on the basis of comorbidities or other clinical characteristics. Archived formalin-fixed, paraffin-embedded (FFPE) tissue blocks from these patients' primary tumors were retrieved from the pathology laboratory archives of the Department of Pathology of the hospital and assessed for PD-L1 expression by immunohistochemistry.

Patient demographics, perioperative data, and postoperative clinicopathological data were collected through retrospective review of medical records and histopathology reports. Demographic characteristics included gender and age at the time of surgery, with patients categorized into two age groups: <55 years and ≥55 years. Perioperative data comprised preoperative clinical impression, extent of surgery (hemithyroidectomy or total thyroidectomy), performance of lymph node dissection and/or laryngectomy, and surgical complications (if any). Postoperative clinicopathological data included tumor characteristics such as maximum tumor diameter, pT stage, lymph node involvement (pN stage), number and location of metastatic lymph nodes, presence of distant metastasis (M stage), tumor-node-metastasis (TNM) stage, histologic subtype, and presence and type of aggressive histological variants. Tumor staging (pT, pN, M, and TNM) was based on the 8th edition (2017) of the American Joint Committee on Cancer (AJCC) TNM classification [15]. For tumors originally staged according to the 7th or previous edition, restaging was performed by an experienced, independent pathologist using core data from the original pathology reports. Additional features recorded were tumor laterality, multifocality, extrathyroidal extension, vascular invasion, perineural invasion, and coexisting Hashimoto’s thyroiditis. Extrathyroidal extension was recorded as present when either microscopic or gross invasion beyond the thyroid capsule was documented in the final histopathology report. The use of postoperative RAI therapy was also recorded. Finally, follow-up data, including disease recurrence and patient vital status, were also obtained through medical record review to assess clinical outcomes. Vital status was determined based on the last documented follow-up visit in the medical records. If follow-up data were missing or outdated (i.e., more than 12 months before the time of data collection), vital status was ascertained through the national death registry using the patient’s social security number.

The study was conducted in accordance with the principles of the Declaration of Helsinki and all applicable local regulations. Ethical approval was granted by the Institutional Review Board of the hospital. As this was a retrospective study using archived FFPE tissue specimens, the requirement for informed consent was waived. All patient data and tissue specimens were pseudoanonymized before analysis to ensure confidentiality. 

IHC staining

The FFPE tumor tissue blocks were cut into 4-μm sections (two per patient), mounted on positively charged glass slides, and then placed in an oven at 58°C ± 2°C for 1 hour. PD-L1 IHC staining was performed using the FDA-approved PD-L1 IHC 22C3 pharmDx assay (Agilent Technologies, Inc., Santa Clara, CA, USA) on the Dako Autostainer Link 48 (code AS480), according to the manufacturer’s instructions [16]. Briefly, sections were pretreated using a 3-in-1 procedure (deparaffinization, rehydration, and target retrieval) in the PT Link system (code PT100/PT101/PT200) with the EnVision™ FLEX Target Retrieval Solution and low pH (1× working solution; code K8005) at 97 °C for 20 minutes. Endogenous peroxidase activity was blocked for 5 minutes with the EnVision FLEX Peroxidase-Blocking Reagent. Slides were then incubated for 30 minutes using the ready-to-use anti-PD-L1 22C3 monoclonal mouse primary antibody, followed by the EnVision FLEX+ Mouse LINKER for 10 minutes and the EnVision FLEX HRP visualization reagent for 30 minutes. The enzymatic conversion of the subsequently added 3,3’-diaminobenzidine tetrahydrochloride (DAB) chromogen for 10 minutes, followed by the addition of DAB enhancer for 5 minutes, resulted in the precipitation of a visible reaction product at the site of the antigen. Between steps, slides were washed with EnVision FLEX Wash Buffer (1× working solution; code K8007). Finally, sections were counterstained with hematoxylin (code K8008), dehydrated in ethanol, cleared in xylene, and coverslipped. Each IHC run included a positive control (tonsil tissue) and a negative control (a ready-to-use monoclonal mouse IgG antibody provided in the PD-L1 IHC 22C3 pharmDx kit).

PD-L1 expression was then assessed by an experienced, independent pathologist blinded to clinicopathological data using a light microscope (20× magnification). Scoring was based on the combined positive score (CPS), calculated as the number of PD-L1-positive cells (including tumor cells, lymphocytes, and macrophages) divided by the total number of viable tumor cells, multiplied by 100. Convincing partial or complete linear membrane staining (≥1+) of viable tumor cells, perceived as distinct from cytoplasmic staining, was scored as positive, as was convincing membrane and/or cytoplasmic staining (≥1+) of lymphocytes and macrophages within tumor nests and/or immediately adjacent supporting stroma. A minimum of 100 viable tumor cells per tissue section was required for the section to be considered adequate for PD-L1 evaluation. A CPS ≥10 was considered positive [16,17].

Statistical analysis

No a priori sample size calculation was performed, as the study was retrospective in nature and based on available medical records. To ensure feasibility while maintaining representativeness, a random sample of 100 patients with a histopathological diagnosis of PTC during the study period was selected. Descriptive statistics were primarily used to summarize the data. Categorical variables were reported as relative and absolute frequencies, while continuous variables were summarized based on the number of available observations (n), mean, standard deviation, median, minimum, and maximum. All variables are presented both overall and stratified by PD-L1 CPS, using a cut-off of 10. To examine associations, the independent t-test and chi-square test were applied when statistical assumptions were met; otherwise, the Wilcoxon rank-sum test and Fisher’s exact test were used. Disease-free survival (DFS) was defined as the time from surgery until the first documented disease recurrence, while overall survival (OS) was defined as the time from surgery until death from any cause. Patients who were alive and recurrence-free at the time of analysis were censored at the date of last available information, as determined from medical records or national registry data. Only deaths confirmed to be the result of the disease were counted as events; other deaths were censored at the time of death. Time-to-event outcomes were estimated using the Kaplan-Meier method, and differences in DFS and OS by CPS status were assessed using the log-rank test. Statistical analysis was performed using R version 4.4.3 (R Foundation for Statistical Computing, Vienna, Austria). The level of statistical significance was set at α=0.05.

## Results

Patient demographics and perioperative characteristics

Of the 100 randomly selected patients, 19 were excluded due to missing or incomplete clinical or histopathological data (n=9) or unavailability of adequate FFPE tissue blocks for PD-L1 assessment (n=10). Thus, a total of 81 patients with a histopathologically confirmed diagnosis of PTC following thyroid surgery between 2007 and 2018 were included in the study. Key patient demographic and perioperative characteristics are summarized in Table 1. Patients were predominantly female (61/81 (75.3%)), and the median age at the time of surgery was 48 years (range: 22-75 years), with the majority (57/81 (70.4%)) being under the age of 55 years.

The most common preoperative clinical impression was thyroid carcinoma, either localized (28/81 (34.6%)) or with suspected metastatic disease (13/81 (16.0%)); the latter often first identified through a malignant cervical lymph node. Other indications for thyroid surgery included multinodular goiter (23/81 (28.4%)), solitary thyroid nodule (11/81 (13.6%)), substernal goiter (3/81 (3.7%)), and Graves’ disease (3/81 (3.7%)).

All patients underwent total thyroidectomy. Concomitant lymph node dissection was performed in 21/81 (25.9%) patients, all involving the central compartment, with or without lateral compartment dissection (11/81 (13.6%) and 10/81 (12.3%), respectively). Laryngectomy was performed in only 2/81 (2.5%) patients. Most patients did not experience surgical complications (58/81 (71.6%)), while transient hypocalcemia occurred in 17/81 (21.0%), transient recurrent laryngeal nerve paralysis in 5/81 (6.2%), and permanent hypocalcemia in 1/81 (1.2%) (Table 1).

**Table 1:** Patient demographic and perioperative clinical characteristics. All data are presented as n (%) unless stated otherwise. RLN, Recurrent laryngeal nerve.

Characteristic	Overall (N=81)
Gender	
Male	20 (24.7%)
Female	61 (75.3%)
Age at surgery (years), median (range)	48.00 (22.0–75.0)
<55	57 (70.4%)
≥55	24 (29.6%)
Preoperative clinical impression	
Thyroid carcinoma	28 (34.6%)
Metastatic thyroid carcinoma	13 (16.0%)
Solitary thyroid nodule	11 (13.6%)
Multinodular goiter	23 (28.4%)
Substernal goiter	3 (3.7%)
Graves' disease	3 (3.7%)
Lymph node dissection	
No	60 (74.1%)
Yes	21 (25.9%)
Central (Level VI) only	10 (12.3%)
Central (Level VI) + ipsilateral lateral (Levels II–V)	8 (9.9%)
Central (Level VI) + bilateral lateral (Levels II–V)	3 (3.7%)
Laryngectomy	
No	79 (97.5%)
Yes	2 (2.5%)
Surgical complications	
Transient hypocalcemia	17 (21.0%)
Permanent hypocalcemia	1 (1.2%)
Transient RLN paralysis	5 (6.2%)
No complications	58 (71.6%)

Clinicopathological characteristics and association with PD-L1 expression

Based on postoperative histopathological examination, the median primary tumor size was 1.00 cm (range: 0.2-7.0 cm), with tumors in 51/81 (63.0%) patients classified as pT1-pT2, indicating confinement to the thyroid gland. In contrast, extrathyroidal extension was identified in 29/81 (35.8%) patients. Most patients had unifocal disease (49/81 (60.5%)), with the number of tumor foci ranging from 1 to 7 (median: 1), and bilaterality was observed in 18/81 (22.2%) patients. Lymph node metastases were present in 18/81 (22.2%) patients, with 6/81 (7.4%) having central compartment involvement only (N1a), while 12/81 (14.8%) had both central and lateral compartment involvement, corresponding to N1b staging. Distant metastasis to the lungs (M1) was documented in a single patient (1/81 (1.2%)). According to the 8th edition of the AJCC TNM staging system [15], the vast majority of patients had early-stage cancer (78/81 (96.3%)), with 69/81 (85.2%) being at stage I, 9/81 (11.1%) at stage II, and only 3/81 (3.7%) at stages III-IVB. Vascular or perineural invasion was absent in most patients (77/81 (95.1%)) (Table 2).

The predominant histologic subtype was follicular (36/81 (44.4%) patients), followed by mixed papillary-follicular (33/81 (40.7%) patients) and classical papillary carcinoma (12/81 (14.8%) patients). Aggressive histologic variants, including tall cell, columnar cell, hobnail, solid, and diffuse sclerosing patterns, were identified in 18/81 (22.2%) patients. Other concurrent thyroid neoplasms were rare, observed in only 5/81 (6.2%) patients. Coexisting Hashimoto’s thyroiditis was present in 23/81 (28.4%) patients.

PD-L1 positivity (CPS ≥10) was observed in 37 (45.7%) of the total cohort. Stratification by PD-L1 status revealed several significant clinicopathological differences between the CPS ≥10 and CPS <10 groups. In detail, tumors in the CPS ≥10 group were significantly larger (median: 1.20 cm vs. 0.50 cm, p=0.002) and more frequently associated with advanced primary tumor stage. Specifically, 24/37 (64.9%) patients in the CPS ≥10 group were classified as pT3-pT4, compared to only 6/44 (13.6%) patients in the CPS <10 group (p<0.001), whereas pT1-pT2 tumors were more common in the CPS <10 group (38/44 (86.4%) vs. 13/37 (35.1%)). CPS ≥10 also demonstrated higher rates of extrathyroidal extension (24/37 (64.9%) vs. 5/44 (11.4%), p<0.001) and lymph node metastasis (17/37 (45.9%) vs. 1/44 (2.3%), p<0.001), with significantly higher frequency of lateral compartment involvement (N1b: 11/37 (29.7%) vs. 1/44 (2.3%)).

Aggressive histologic variants were substantially overrepresented in the CPS ≥10 group (16/37 (43.2%) vs. 2/44 (4.5%), p<0.001). Although bilaterality (11/37 (29.7%) vs. 7/44 (15.9%)) and multifocality (17/37 (45.9%) vs. 15/44 (34.1%)) were more frequent in the CPS ≥10 group, these differences did not reach statistical significance (p=0.14 and p=0.2, respectively). However, a significantly greater proportion of patients in the CPS ≥10 group had ≥4 tumor foci (9/37 (24.3%) vs. 2/44 (4.5%), p=0.034). Coexisting Hashimoto’s thyroiditis was also more prevalent among patients with CPS ≥10 (17/37 (45.9%) vs. 6/44 (13.6%), p<0.001). Finally, RAI was more commonly administered in patients in the CPS ≥10 group (30/37 (81.1%) vs. 14/44 (31.8%), p<0.001), consistent with the higher-risk pathological features observed. A more detailed comparison of clinicopathological features by PD-L1 CPS status is presented in Table 2.

**Table 2:** Demographic and clinicopathological characteristics of patients overall and by PD-L1 expression status. ^1^Fisher's exact test or Pearson's chi-square test. ^2^In unifocal disease, tumor size refers to the primary tumor and in multifocal to the foci with the maximum size. All data are presented as n (%) unless stated otherwise. Bold indicates statistical significance at p<0.05. CPS, Combined positive score; LN, Lymph node; PD-L1, Programmed cell death ligand 1; RAI, Radioactive iodine; TNM, Tumor-node-metastasis.

Characteristic	Overall (N=81)	CPS <10 (N=44)	CPS ≥10 (N=37)	p-value^1^
Gender				0.60
Male	20 (24.7%)	12 (27.3%)	8 (21.6%)	
Female	61 (75.3%)	32 (72.7%)	29 (78.4%)	
Age at surgery (years), median (range)	48.00 (22.0–75.0)	50.50 (22.0–71.0)	43.00 (27.0–75.0)	0.10
Tumor size (cm), median (range)	1.00 (0.2–7.0)	0.50 (0.2–4.5)	1.20 (0.3–7.0)	0.002
pT stage				<0.001
pT1–pT2	51 (63.0%)	38 (86.4%)	13 (35.1%)	
pT3–pT4	30 (37.0%)	6 (13.6%)	24 (64.9%)	
LN metastasis (pN stage)				
N0	63 (77.8%)	43 (97.7%)	20 (54.1%)	<0.001
N1	18 (22.2%)	1 (2.3%)	17 (45.9%)	
N1a	6 (7.4%)	0 (0.0%)	6 (16.2%)	
N1b	12 (14.8%)	1 (2.3%)	11 (29.7%)	
Number of involved LNs, median (range)	6.00 (1.0–22.0)	4.00 (4.0–4.0)	6.00 (1.0–22.0)	0.6
Location of involved LNs				>0.9
Central	5 (27.8%)	0 (0.0%)	5 (29.4%)	
Central + lateral	13 (72.2%)	1 (100.0%)	12 (70.6%)	
Distant metastasis (M stage)				0.5
M0	80 (98.8%)	44 (100.0%)	36 (97.3%)	
M1	1 (1.2%)	0 (0.0%)	1 (2.7%)	
TNM stage				0.590
I–II	78 (96.3%)	43 (97.7%)	35 (94.6%)	
III–IVB	3 (3.7%)	1 (2.3%)	2 (5.4%)	
Histologic subtype				0.13
Classic papillary	12 (14.8%)	5 (11.4%)	7 (18.9%)	
Mixed papillary follicular	33 (40.7%)	15 (34.1%)	18 (48.6%)	
Follicular	36 (44.4%)	24 (54.5%)	12 (32.4%)	
Aggressive variant				<0.001
No	63 (77.8%)	42 (95.5%)	21 (56.8%)	
Yes	18 (22.2%)	2 (4.5%)	16 (43.2%)	
Solid variant	1 (1.2%)	0 (0.0%)	1 (2.7%)	
Diffuse sclerosing variant	4 (4.9%)	0 (0.0%)	4 (10.8%)	
Hobnail variant	2 (2.5%)	0 (0.0%)	2 (5.4%)	
Columnar cell variant	3 (3.7%)	1 (2.3%)	2 (5.4%)	
Tall cell	7 (8.6%)	1 (2.3%)	6 (16.2%)	
Tall cell + hobnail variant	1 (1.2%)	0 (0.0%)	1 (2.7%)	
Laterality				0.14
Unilateral	63 (77.8%)	37 (84.1%)	26 (70.3%)	
Bilateral	18 (22.2%)	7 (15.9%)	11 (29.7%)	
Focality				0.2
Unifocal	49 (60.5%)	29 (65.9%)	20 (54.1%)	
Multifocal	32 (39.5%)	15 (34.1%)	17 (45.9%)	
Number of foci, median (range)	1.00 (1.0-7.0)	1.00 (1.0- 4.0)	1.00 (1.0-7.0)	0.076
1	49 (60.5%)	29 (65.9%)	20 (54.1%)	0.034
2-3	21 (25.9%)	13 (29.5%)	8 (21.6%)	
4+	11 (13.6%)	2 (4.5%)	9 (24.3%)	
Extrathyroidal extension				<0.001
Absent	52 (64.2%)	39 (88.6%)	13 (35.1%)	
Present	29 (35.8%)	5 (11.4%)	24 (64.9%)	
Vascular invasion				>0.9
Absent	77 (95.1%)	42 (95.5%)	35 (94.6%)	
Present	4 (4.9%)	2 (4.5%)	2 (5.4%)	
Perineural invasion				>0.9
Absent	77 (95.1%)	42 (95.5%)	35 (94.6%)	
Present	4 (4.9%)	2 (4.5%)	2 (5.4%)	
Coexisting thyroid neoplasm				0.4
No	76 (93.8%)	40 (90.9%)	36 (97.3%)	
Yes	5 (6.2%)	4 (9.1%)	1 (2.7%)	
Coexisting Hashimoto’s thyroiditis				<0.001
No	58 (71.6%)	38 (86.4%)	20 (54.1%)	
Yes	23 (28.4%)	6 (13.6%)	17 (45.9%)	
Postoperative RAI				<0.001
No	37 (45.7%)	30 (68.2%)	7 (18.9%)	
Yes	44 (54.3%)	14 (31.8%)	30 (81.1%)	

**Figure 1:**
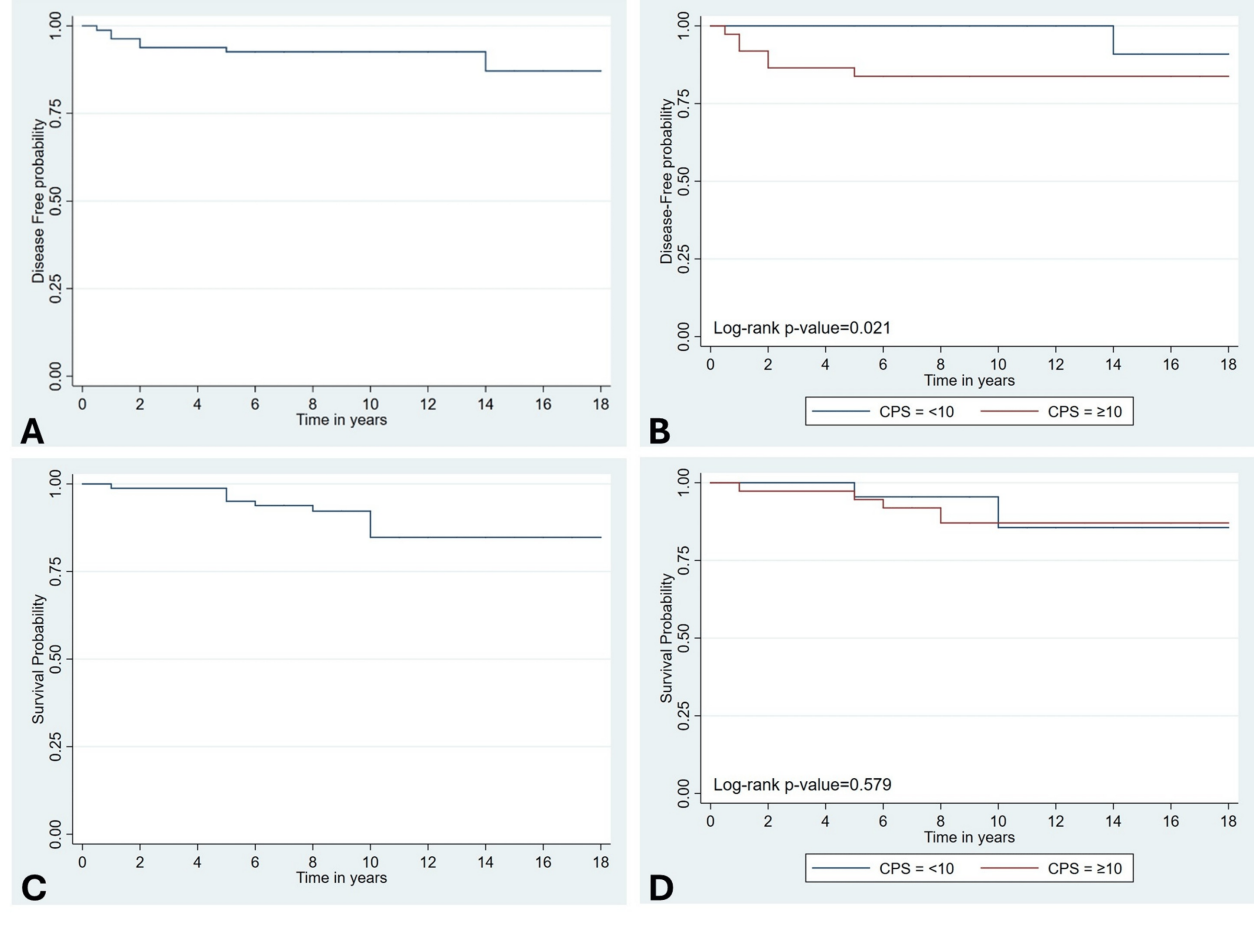
Kaplan-Meier analysis of (A) DFS in the overall cohort, (B) DFS stratified by PD-L1 expression (CPS <10 vs. CPS ≥10), (C) OS in the overall cohort, and (D) OS by PD-L1 expression (CPS <10 vs. CPS ≥10). CPS, Combined positive score; DFS, Disease-free survival; OS, Overall survival; PD-L1, Programmed cell death ligand 1.

At a median follow-up of nine years, disease recurrence occurred in 7/81 (8.6%) patients, being significantly more frequent in the CPS ≥10 group (6/37 (16.2%) vs. 1/44 (2.3%), p=0.043). One patient in the CPS ≥10 group died due to distant (lung) metastases, representing the only disease-specific mortality in the cohort. Management of recurrent disease in the CPS ≥10 group involved lateral lymph node dissection in 3/6 (50.0%) patients and RAI therapy in 2/6 (33.3%) patients (Table 3). As shown in the Kaplan-Meier curve (Figure 1A), the median DFS was not reached (NR) in the overall cohort. Notably, all recurrence events occurred within the first 18 months of follow-up. When stratified by PD-L1 expression (Figure 1B), patients with CPS ≥10 experienced significantly shorter DFS compared to those with CPS <10 (log-rank p=0.021), although the median DFS was NR in either group. OS remained high across the study cohort (Figure 1C), consistent with the generally favorable prognosis of PTC. Among the nine deaths recorded (9/81 (11.1%)), eight were non-disease-related (five in the CPS <10 group (5/44 (11.4%)) and three in the CPS ≥10 group (3/37 (8.1%)). No significant difference in OS was observed between CPS subgroups (Figure 1D; log-rank p=0.579), and the median OS was NR in either group.

**Table 3:** Follow-up data overall and by PD-L1 expression status. ^1^Fisher's exact test. ^2^Percentages calculated among patients with recurrence (N=7 overall; N=1 in CPS <10; N=6 in CPS ≥10). All data are presented as n (%) unless stated otherwise. Bold indicates statistical significance at p<0.05. CPS, Combined positive score; LN, Lymph node; PD-L1, Programmed cell death ligand 1; RAI, Radioactive iodine.

	Overall (N=81)	CPS <10 (N=44)	CPS ≥10 (N=37)	p-value^1^
Follow-up (years)	9	12	8	
Recurrence				<0.043
No	74 (91.4%)	43 (97.7%)	31 (83.8%)	
Yes	7 (8.6%)	1 (2.3%)	6 (16.2%)	
Management of recurrence^2^				
Death	1 (16.7%)	0 (0.0%)	1 (16.7%)	
LN dissection (levels II–V)	2 (33.3%)	0 (0.0%)	2 (33.3%)	
LN dissection (levels II–VI)	1 (16.7%)	0 (0.0%)	1 (16.7%)	
RAI	2 (33.3%)	0 (0.0%)	2 (33.3%)	
Missing	1	1	0	

## Discussion

The expression of PD-L1 is a well-established prerequisite for the use of immune checkpoint inhibitors and has been increasingly recognized and used as a prognostic biomarker in several malignancies [9]. However, its prognostic significance in TC, particularly PTC, remains largely unexplored and subject to ongoing debate. This study seeks to address this gap by analyzing PD-L1 expression, as assessed using the CPS, and investigating its association with key clinicopathological features in a cohort of 81 Greek patients with PTC.

In terms of overall clinical characteristics, patients in this cohort were predominantly female (75.3%) with a median age of 48 years, consistent with the known epidemiology of PTC, which is more common in women and typically diagnosed in the fourth to fifth decades of life [1,2]. All patients underwent total thyroidectomy, and about one-quarter also received central and/or lateral neck dissection, in line with local surgical practice patterns in thyroid oncology in Greece. Most patients had early-stage PTC (stages I-II), and all but one had no evidence of distant metastasis at the time of surgery. Nevertheless, a considerable proportion had features of more aggressive disease, including extrathyroidal extension (35.8%), nodal metastases (22.2%), and aggressive histologic variants (22.2%). Hashimoto’s thyroiditis was also present in over one-quarter of patients. These characteristics reflect a predominantly low- to intermediate-risk PTC population [18] while still capturing the clinical heterogeneity of the disease.

Notably, nearly half of our cohort (45.7%) exhibited PD-L1 positivity (CPS ≥10), a prevalence falling in the middle of the broad range reported in the literature (6.1% to 82.5%) [19-27]. PD-L1 positivity was significantly associated with larger tumor size and higher pT stage, extrathyroidal extension, lymph node metastasis, and the presence of aggressive histologic variants. These associations support a link between PD-L1 expression and biologically aggressive tumor features, consistent with similar observations in several earlier studies [9,20,23,28], although not universally reported [19,21,22,25,27]. Importantly, our study is among the few to investigate the association between PD-L1 expression and aggressive histologic variants. In fact, variants such as tall cell, columnar, hobnail, and diffuse sclerosing were markedly overrepresented in the CPS ≥10 group, a finding not observed by Bai et al. but consistent with more recent data from Siraj et al., who specifically reported a higher frequency of the tall cell variant among PD-L1-positive patients [22,24,26]. Given that these variants are independently associated with poorer outcomes, their co-occurrence with high PD-L1 expression may help define a biologically distinct, higher-risk subgroup within PTC.

A significant association was also identified between PD-L1 expression and coexisting Hashimoto’s thyroiditis, in line with findings from previous studies and confirmed by meta-analyses [12,13,20,22,23,25,27,28]. PD-L1 expression has also been observed in Hashimoto’s thyroiditis in the absence of PTC, supporting the notion that autoimmune inflammation may independently drive PD-L1 upregulation. Indicatively, Lubin et al. revealed that PD-L1 was upregulated not only in PTC cells but also in non-neoplastic follicular epithelium in the background of Hashimoto’s thyroiditis, suggesting an adaptive immune response, in which PD-L1-negative follicular cells are eliminated by the autoimmune infiltrate, leaving behind a PD-L1-enriched cell population [28]. Chowdhury et al. further proposed that the inflammatory microenvironment, rich in cytokines such as interferon gamma, IL-1, IL-6, and IL-10, could trigger PD-L1 upregulation, potentially inducing thyroid tumorigenesis [25]. While these mechanisms remain speculative, they provide biologically plausible explanations for our findings. Further mechanistic research is needed to elucidate the immunologic interplay between thyroiditis and PD-L1 expression in PTC.

At a median follow-up of nine years, disease recurrence was observed in 8.6% of our cohort, which falls within the known ranges for low- to intermediate-risk PTC populations [18]. Recurrence was significantly more common among patients with CPS ≥10, and Kaplan-Meier analysis demonstrated a shorter DFS in this group. Similar associations between PD-L1 expression, recurrence, and reduced DFS have been reported in meta-analyses of differentiated TC, particularly in PTC [12-14]. Collectively, these findings suggest that PD-L1 may serve as a biomarker of early recurrence and more aggressive clinical behavior in PTC. However, given the limited number of recurrence events in our cohort, these observations should be interpreted with caution and validated in larger, prospective cohorts. If validated in future prospective studies, PD-L1 expression could potentially aid in refining postoperative risk stratification and guiding more tailored surveillance strategies in patients with PTC.

Interestingly, patients with PD-L1-positive tumors were more likely to receive adjuvant RAI therapy. While PD-L1 status was not used to guide treatment, this likely reflects the association of PD-L1 expression with high-risk pathological features that influence adjuvant decision-making. Although this observation does not establish a direct clinical role for PD-L1, it highlights the relevance of PD-L1-positive status in current management patterns and supports its further evaluation as a potential marker for individualized risk stratification.

Discrepancies in reported PD-L1 expression and its clinicopathological associations across studies may stem from variability in tissue preparation, IHC assays, PD-L1 antibody clones, scoring systems, cut-off values, and interpretation criteria [9,12,29]. Unlike other malignancies (e.g., lung, breast, and urothelial), no consensus currently exists on the optimal scoring approach in TC. While most prior studies have used the tumor proportion score (TPS), typically applying cut-offs of ≥1% and 5% [19-22,26,27], we employed the CPS, which incorporates both tumor and immune cell staining, thereby better reflecting the tumor immune microenvironment. While not routinely applied in TC, CPS is widely adopted in other tumor types, and a threshold of ≥10 has been proposed as more likely to capture biologically relevant expression and identify patients most likely to benefit from immunotherapy [16,17].

Further heterogeneity arises from the use of different antibody clones (e.g., E1L3N, SP142, 22C3, SP263, and Ab174838), each with differing sensitivity and staining patterns. SP142, in particular, has shown significantly lower sensitivity in PD-L1 tumor cell staining, which may partially explain the lowest PD-L1 positivity rate (6.1%) reported in PTC by Ahn et al. [19,29], further compounded by their use of tissue microarrays, instead of whole tissues, that may underestimate intratumoral heterogeneity [29]. Interpretation in terms of subcellular localization further complicates PD-L1 assessment, with higher expression rates being reported when cytoplasmic, rather than membranous, staining is considered positive. For example, Cunha et al. reported an 82.5% positivity rate, among the highest to date in PTC, which may in part reflect their inclusion of cytoplasmic staining in their scoring [29,30]. However, only PD-L1 expressed on the cell membrane is capable of engaging PD-1 on immune cells to mediate immune suppression. Accordingly, clinical trials evaluating anti-PD-1/PD-L1 therapies have based patient selection on membranous staining, and there is a growing consensus that cytoplasmic staining should not be interpreted as positive [9]. These methodological inconsistencies may partly explain why PD-L1 has not yet been firmly established as a prognostic marker in PTC, underscoring the need for standardization of PD-L1 assessment methods. In our study, we used the 22C3 clone on the pharmDx platform, an FDA-approved and clinically validated assay that specifically detects membranous PD-L1 expression. Furthermore, whole-tissue sections were used to account for intratumoral heterogeneity, and scoring was restricted to membranous expression, aligning with current standards in immuno-oncology.

Our study has several limitations. First, although patients were randomly sampled, eligibility required the availability of adequate tissue material and complete clinical and histopathological data. This may have inadvertently introduced selection bias by excluding patients with incomplete records or insufficient specimens, thereby limiting the representativeness of the broader PTC population treated in our hospital. In addition, the retrospective and observational nature of the study precludes causal inference, so the associations identified should be regarded as exploratory and hypothesis-generating, rather than definitive. The absence of multivariate adjustment further limits interpretation, as it precludes assessment of whether PD-L1 expression is independently associated with adverse features or recurrence, or whether the observed associations are confounded by established prognostic factors such as tumor size, stage, extrathyroidal extension, nodal status, or histologic subtype. External validity is also limited. The single-center setting and modest sample size restrict generalizability, as the cohort may not capture the full spectrum of patients with PTC encountered in more diverse clinical settings. The ethnically homogeneous Greek population further narrows applicability, while the predominance of early-stage disease may underestimate the potential relevance of PD-L1 expression in advanced PTC, where immune evasion mechanisms are expected to play a greater role. Although the median follow-up was sufficient to capture early recurrences, the lack of active, prospective follow-up and reliance solely on medical record review may have resulted in under-detection of late or asymptomatic events, particularly given the typically indolent course of PTC. The limited number of recurrence and mortality events further reduces the statistical power to draw firm prognostic conclusions. Additionally, due to the retrospective nature of the study and variability in documentation, we were unable to systematically distinguish between structural and biochemical recurrence. While a subset of patients received adjuvant RAI or lymph node dissection during follow-up, the indication (e.g., rising thyroglobulin vs. imaging findings) was not consistently available. Of note, serum thyroglobulin levels were not included in our data collection process, as they were not uniformly documented in the medical records. This precluded further evaluation of biochemical recurrence and potential associations with PD-L1 expression. Prospective studies with standardized data collection would be better positioned to address such aspects. Moreover, PD-L1 expression was assessed using CPS ≥10, a threshold not yet validated in TC; while considered appropriate for the objectives of this study, exploring additional thresholds or alternative scoring systems (e.g., TPS) could yield further insights into PD-L1 expression dynamics and prognostic relevance. Lastly, molecular alterations such as BRAF mutations were not assessed, as their evaluation was beyond the scope and resources of this study. This omission limits further interpretation of potential interactions between PD-L1 expression and oncogenic drivers in PTC. 

Despite these limitations, to our knowledge, this is the first study to provide real-world data on PD-L1 expression in a well-defined cohort of patients with PTC in Greece, thereby contributing to the limited literature on this topic. Methodological rigor was ensured through the use of an FDA-approved, clinically validated IHC assay, blinded scoring, and detailed clinicopathological information, which enhances the robustness of our exploratory findings and highlights their potential clinical significance. However, to better understand the independent prognostic role of PD-L1 and establish its clinical utility, future research should ideally include larger, prospective, multicenter cohorts with extended follow-up, integration of molecular profiling, and multivariate analyses.

## Conclusions

Our study underscores a potential association between PD-L1 expression and adverse clinicopathological features in PTC. While these findings may support the relevance of PD-L1 as a surrogate biomarker of tumor aggressiveness and suggest its potential utility in risk stratification, they should be interpreted with caution, given the study’s limitations, including its retrospective design, limited sample size, and inability to support causal inferences. Looking ahead, future prospective, multicenter studies with larger cohorts are warranted to validate these findings and further elucidate the prognostic and therapeutic relevance of targeting the PD-1/PD-L1 axis in appropriately selected PTC populations.
